# Adaptation Strategies in Students with Motor Functional Diversity* **

**DOI:** 10.17533/udea.iee.v41n1e13

**Published:** 2023-03-14

**Authors:** Maribel Franco Buitrago, Luisa María Muñoz Jara, Nicol Dayana Vargas Pérez, Nancy García García

**Affiliations:** 1 Undergraduate nursing student. Research Group: GRIEEQ, Universidad del Quindío, Colombia. Email: maribel.francob@uqvirtual.edu.co. Universidad del Quindío Universidad del Quindío Colombia maribel.francob@uqvirtual.edu.co; 2 Undergraduate nursing student. Research Group: GRIEEQ, Universidad del Quindío, Colombia. Email: luisam.munozj@uqvirtual.edu.co Universidad del Quindío Universidad del Quindío Colombia luisam.munozj@uqvirtual.edu.co; 3 Undergraduate nursing student. Research Group: GRIEEQ, Universidad del Quindío, Colombia. Email: nicold.vargasp@uqvirtual.edu.co Universidad del Quindío Universidad del Quindío Colombia nicold.vargasp@uqvirtual.edu.co; 4 Nurse, M.Sc. Profesora. Research Group: GRIEEQ, Universidad del Quindío, Colombia. Email: ngarcia@uniquindio.edu.co Universidad del Quindío Universidad del Quindío Colombia ngarcia@uniquindio.edu.co

**Keywords:** motor disorders, disabled persons, emotional adjustment, health strategies, students, universities, qualitative research, trastornos motores, personas con discapacidad, ajuste emocional, estrategias de salud, estudiantes, universidades, investigación cualitativa, transtornos motores, pessoas com deficiência ajustamento emocional, estratégias de saúde, estudantes, pesquisa qualitativa

## Abstract

**Objective.:**

The work sough to know the adaptation strategies of students from Universidad del Quindío with motor functional diversity.

**Method.:**

Descriptive qualitative study with a phenomenological approach. Data were collected through an in-depth interview with nine undergraduate students with moderate motor functional diversity, in face-to-face class attendance modality during the period 2022-2 at Universidad del Quindío (Colombia) with age ≥ 18 years and having scored from 20 - 40 in the Barthel index. The definition of the number of participants was conducted through theoretical saturation.

**Results.:**

Seven categories emerged from the descriptive analysis of the interviews: 1) support; 2) affection; 3) life project; 4) personal growth; 5) spirituality; 6) autonomy, and 7) education. Together, they reveal important aspects on the way students have adapted to the university campus and how interpersonal relations can contribute to promoting resilience processes.

**Conclusion.:**

Support and affection provided by the social setting play a fundamental role in the adaptation of students with motor functional diversity, improving their mental health, generating resilience, and increasing their self-esteem. Noting that in spite of lifestyle changes after the acquisition of the diversity, the students set novel goals and develop new abilities that contribute to complying with their life project; likewise, they have set into practice and can recognize their coping mechanisms, acquiring qualities, like resilience and autonomy.

## Resumen

**Objetivo.:**

Conocer las estrategias de adaptación de los estudiantes con diversidad funcional motora de la universidad del Quindío.

**Método.:**

Estudio cualitativo de tipo descriptivo con enfoque fenomenológico. Los datos se recolectaron a través de una entrevista a profundidad con 9 estudiantes de pregrado con diversidad funcional motora moderada, en modalidad presencial en el período 2022-2 de la Universidad del Quindío (Colombia) con una edad igual o mayor a 18 años y haber puntuado de 20-40 en el índice de Barthel. La definición del total de participantes se realizó por saturación teórica.

**Resultados.:**

Del análisis descriptivo de las entrevistas surgieron siete categorías: 1) apoyo; 2) afecto; 3) proyecto de vida; 4) crecimiento personal; 5) espiritualidad; 6) autonomía y 7) educación. En conjunto, revelan aspectos importantes sobre la forma en que los estudiantes se han adaptado al campus universitario y cómo las relaciones interpersonales pueden contribuir a la promoción de procesos de resiliencia.

**Conclusión.:**

El apoyo y el afecto brindado por el entorno social juega un papel fundamental en la adaptación de los estudiantes con diversidad funcional motora, mejorando su salud mental, generando resiliencia y aumentando su autoestima. Constatando que a pesar de los cambios en el estilo de vida tras la adquisición de la diversidad los alumnos se plantean novedosas metas y desarrollan nuevas habilidades que contribuyen al cumplimiento de su proyecto de vida, así mismo, han puesto en práctica y pueden reconocer sus mecanismos de afrontamiento adquiriendo cualidades como la resiliencia y la autonomía.

## Resumo

**Objetivo.:**

Conheça as estratégias de adaptação de alunos com diversidade motora funcional na Universidade de Quindío, (Colômbia).

**Método.:**

Estudo qualitativo do tipo descritivo com abordagem fenomenológica. Os dados foram recolhidos através de entrevista em profundidade a 9 alunos de graduação com moderada diversidade motora funcional, na modalidade presencial no período 2022-2, com idade igual ou superior a 18 anos e com pontuação 20-40 no índice de Barthel. A definição do número total de participantes foi feita por saturação teórica.

**Resultados.:**

Da análise descritiva das entrevistas emergiram sete categorias: 1) apoio; 2) afeto; 3) projeto de vida; 4) crescimento pessoal; 5) espiritualidade; 6) autonomia e 7) educação. Juntos, eles revelam aspectos importantes sobre a forma como os alunos se adaptaram ao campus universitário e como as relações interpessoais podem contribuir para promover processos de resiliência.

**Conclusão.:**

O apoio e o afeto proporcionado pelo meio social têm papel fundamental na adaptação de alunos com diversidade motora funcional, melhorando sua saúde mental, gerando resiliência e aumentando sua autoestima. Constatando que apesar das mudanças no estilo de vida após a aquisição da diversidade, os alunos traçam novas metas e desenvolvem novas habilidades que contribuem para a realização de seu projeto de vida, da mesma forma, colocam em prática e conseguem reconhecer seus mecanismos de enfrentamento adquirindo qualidades como resiliência e autonomia.

## Introduction

In 2006, the United Nations Organization defined disability as: A concept that evolves and which results from the interaction between people with deficiencies and barriers due to attitude and the environment, which prevent their full and effective participation in society, on equal terms with others.^1^ Consequently, the words “disability” o “handicap'' have been replaced by “functional diversity”; a new term that refers to the difference, variety, abundance of different things, or dissimilarity.^2^

Moreover, people with functional diversity are subjects of rights, like education; mentioned in article 67 of Colombia’s Political Constitution of 1991, “Education is a right of the person and a public service that bears a social function; with it, access is sought to knowledge, science, technology, and other goods and values of culture”;^3^ further, universities refer to inclusive education as the “overall guiding principle” that seeks to enhance and value diversity, promote respect to being different, and facilitate the community’s participation within an intercultural structure, thus, favoring social cohesion.^4^ Likewise, the universal design for learning applies the concepts of accessibility and inclusion, beyond physical environments, designing teaching and learning opportunities in ways that are varied, accessible, and attractive for all students, even those with different needs,^5^ therefore, it is proposed to create new forms of access to higher education for students with diversity, potentiate ongoing and initial teacher training programs, sensitize the university community, promote participation from students in said condition, establish inclusive public policies, improve accessibility, and disseminate basic information on themes of disability and inclusion.^6^

With regards to the life project, it can change when acquiring a motor functional diversity due to the impact it generates on the parental, cultural, or social roles and assimilating a new environment and these influence directly on how to deal with adaptation;^7^ furthermore, the environment might not have a physical infrastructure that facilitates their mobility in the study campus, as identified by a study that obtained that only one of nine universities in Medellín (Colombia)^8^ had an environment adapted to the needs of students with physical diversity, and this is why a timely adaptation should be conducted of the student to the university campus. 

Bearing in mind that the essence of nursing is that of caring for people, not only within a clinical environment but in any environment where there is the need for care; in this case, the university. As a research group, our objective was to know the adaptation strategies of students with motor functional diversity at Universidad del Quindío, in hand with the adaptation model by Callista Roy^9^ because it allows emphasizing on stimuli to favor an adaptive response, making it important to evaluate the person’s coping mechanisms, given that these are correlated with the effectiveness of their adaptation process,^10^ this will guide us to comprehending their coping strategies and contributing to holistic and differentiated care, promoting disciplinary knowledge.

## Methods

This was a descriptive qualitative study with a phenomenological approach. The population were attending undergraduate students during the second period of 2022 at Universidad del Quindío (Colombia) with moderate motor functional diversity, age ≥ 18 years and having scored from 20 to 40 in the Barthel index. The purposive sampling technique was used to select the participants who complied with the project’s criteria, thus, nine students cooperated and two refused to participate, given that they found the theme uncomfortable; the total definition of the participants was conducted through theoretical saturation.

To operationalize this study, the three researchers did not have any relationship with any student with said criteria, thereby, the participants were found and contacted in the university campus and/or via e-mail through the University Welfare; the informed consent (Annex 1) was provided in person and signed by each of the participants, emphasizing on the voluntary nature of the participation and on the confidentiality. It was clarified that information collected would be used for a research objective. Thereafter, they were asked to respond the Barthel index,^11^ which has a Cronbach’s alpha of 0.86-0.92 with a list of daily activities valued differently, assigning 0, 5, 10, or 15 points. The global range can vary between 0 (completely dependent) and 100 points (completely independent).^12^ Application of this index was carried out with the intention of verifying compliance of the inclusion criteria; then, an interview script was used (Annex 2), related with the three adaptive modes of the relational subsystem of the adaptation model by Callista Roy and with the concept of motor functional diversity, seeking to obtain a broad vision to comprehend and interpret each situation of the participant, that is from their own perception. 

After this, an in-depth interview was conducted in the university campus, lasting a maximum of 90 minutes, which was audio recorded to gather the data and field notes after the interview, with the presence of the researchers and students who reported feeling comfortable and safe during the entire conversation. Thus, to demonstrate the effectiveness of this interview, an exploratory study was conducted with participation of three students and its relevance was evidenced for the fulfillment of the research objectives. The transcriptions of the interviews were revised by the participants who confirmed their content, which is why no interview is repeated. Furthermore, the analysis of the information obtained from the sociodemographic characterization was performed with the SPSS program, version 25, and the processing of the information obtained through the in-depth interviews was conducted with the Atlas. Ti software, version 22,1. 

This research was evaluated by the bioethics committee of the Faculty of Health Sciences at Universidad del Quindío, which granted its support through Act N° 18 of 24 June 2022 (Annex 3) to conduct such. 

## Results

The nine participants were between 20 and 32 years of age, five men and four women; the predominant socioeconomic level was II (56%) and in lower percentage III (44 %); with respect to the work situation, besides studying, 56% are employed. 

**Table 1:** Overall characteristics of the study participants

Participant	Pseudonym	Age	Sex	Socioeconomic level	Occupation
1	Milagros	20 years	Feminine	2	Student
2	Martin	28 years	Masculine	3	Studies and works
3	Javier	30 years	Masculine	3	Studies and works
4	Federico	22 years	Masculine	2	Student
5	Emily	32 years	Feminine	3	Studies and works
6	Joaquín	20 years	Masculine	2	Student
7	Antonia	30 years	Feminine	2	Studies and works
8	Martha	29 years	Feminine	2	Studies and works
9	Eric	23 years	Masculine	3	Student

Seven categories emerged from the descriptive analysis of the interviews: 1) support; 2) affection; 3) life project; 4) personal growth; 5) spirituality; 6) autonomy and 7) education. Together, these reveal important aspects on how the students have adapted to the campus and how interpersonal relations can contribute to promoting resilience processes. Additionally, the results showed that access to the university had a positive impact on the participants’ lives, encouraging them to maintain and/or construct life projects, promoting wellbeing and autonomy. 

### Category 1. Support

The participants expressed that support is the fundamental base for their process of adaptation to the diversity; therein arises the importance of this category: *They play the primordial role, they have it, my family, they are the ones that drive me; you have to do this; we will do this this way; better we do it this way (Martha).*

*Manifestation of support.* Regarding manifestations of support, three settings are often named - social, family, and sentimental, which they consider their “caregivers”: *That month I was in crutches, I stayed at my partner’s house, so it was like everyone kept raising my self-esteem, so I went out a lot, even if I was on crutches I felt that I could do what I did before (Emily).* In addition, they emphasize that the best manifestation of support is that people take the time to learn to recognize their need for care and know how to help without seeing them with pity or shame: *Feeling completely accepted without feeling regret, because some people feel sorry for you, like what a pity; it does more for me to be treated like a normal person, so that makes me feel better, it diminishes my anxiety (Antonia).*

*Professional support.* During the interviews, two professions were mentioned that influenced their adaptation process; the first is psychology, which contributed to their mental health: *The psychologist helped a lot, she also helped me clean, so she told me, every time you talk about your accident, you're cleaning because the brain is not storing the information, but you are freeing yourself (Milagros).* The second profession, and the most named was nursing, which played a fundamental role in mental and physical health, being those who provided companionship mainly during the hospital stay: *A nurse, I remember a lot, she was from the city of Pasto, she gave me a loom, it was on tiptoe, and she gave me the wool and I did not move my hands, she told me… and I still keep it, after 11 years … look, this loom will be good for you, not only to move your hands, it will also help you generate income because you won’t have much, so here I give you this beginning of the process, so enjoy it and I brought the loom with me (Emily); and: A nurse who always put effort, so every day there were exercises and exercises every day, until the moment when I was able to start walking, given the very interest that person delivered and I started to develop my activity and my normal life (Martín).*

### Category 2. Affection

Participants described that on the moment of acquiring the diversity, one of the strategies that helped them most in their adaptation process and resilience were the affection provided by the family and social nucleus: *I feel happy; I feel that wherever I go, I always find affection from many people and I feel that this journey has not been in vain (Martha).*

*Demonstrations of love.* With respect to manifestations of affection by loved ones, the participants report that these are a pillar to face difficult moments during the rehabilitation and adaptation process: *They are a fundamental part because they help you a lot emotionally because it helps to improve yourself as a person and at the moment, they help you to have the strength to face all situations, situations that if you were alone you could have bad thoughts or take bad actions by not having that support (Antonia).*

*Own feelings/emotions.* Feelings are identified as a process, where their initial phase shows a series of negative emotions and frustration: *At the beginning, it is difficult, right?, difficult to accept; you get angry with the world; you don’t want to go out, that happened to me, I don't know if many of us are afraid that people will see us in a wheelchair or with our conditions, acceptance is very difficult (Emily).* However, when developing a proper adaptation process, we evidence that feelings return to a more positive vision, where their greatest characteristic is resilience and empowerment: *I am always thinking positively, I won't say that I'm the happiest, I get up and I never think about problems, that is, I have my difficulties and my things like anyone else has, but I don't think, like on my limitations, no (Federico).* In addition, during the interviews gratitude to life was highlighted for allowing them to once again develop certain activities, thus, evidencing their enthusiasm to live as fully as possible: *I enjoy everything that I can now do, if I have to make coffee, I love doing it because I can move my hands again, I can feel the sensation of drinking it; in a hospital bed I could not do it, so that new opportunity has made me appreciate everything more, everything I am doing again (Emily).*

### Category 3. Life project

This category lets us understand that the individual is a unique being, with defined goals and established dreams, thus, leading the participants to develop resilience to face the change of their life project: *Things are more difficult and complex, but you can adapt to those changes, to those obstacles and difficulties, you can continue your project and your goals (Martín); I changed 100% because before I was an athlete, I was in athletics, I was in the gym, I had already taken courses to be a coach, I was going to start working as a physical trainer and from there the goal was to keep climbing, but then, everything crumbled (Eric); and*, *For me to say that I have not met my objectives, I don't think so, because I am now studying, I work for an entity that I am an example, I’m in a university where when they see me go by, I am also an example, so I am achieving the objectives I want, I am in a place I like to be in, with my work and I am doing my therapy, everything is well balanced, yes, the objectives changed because of my capacities, they are taking a little longer, but I think I'm already fulfilling them and it's never too late to do what you want (Antonia).*

*Change in lifestyle.* The entire population refers to a change in lifestyle; among the most-mentioned aspects there are education, work, and economic field: *I was going to my job, an ordinary girl who studied, worked, normal from home to work, well, normal, and then you can realize how life changes you, in a second - not in minutes - but in seconds (Emily); At first, it was very difficult because when you acquire a diversity you stop working or, for example, in my case that I was working and looking after my son, so you say, how will I live? and you know the state cannot have those types of subsidies, because they don’t exist, there is nothing specific that supports the person with a disability (Martha).*

### Category 4. Personal growth

Regarding personal growth, it was a transformation process of sorts, which implies some obstacles, mostly, with ourselves, considering the most important, self-knowledge and many times to unlearn what is known: *A personal decision of each one, to face what it is like, as a person physically, as well as the conditions that you have and that is what makes you change your - navigation - route (Javier).*

*Experience of diversity.* All the participants acquired the motor functional diversity between the youth and adulthood life cycles due to multiple causes: *I had a car accident when entering the city of Pereira, I fractured the head of the femur in my left leg, I had surgery in which they placed three cannulated titanium screws (Federico). There was a fight in the bus, but it was not with me, a claim made by a user with another and in that discrepancy, a stray bullet lodged in the part of the neck, it affected this whole side, then my head was left to one side, I was left with a diagnosis of quadriplegia, the diagnosis itself was not good, I could only communicate through my eyes because the air I had for my lungs I had to reserve for breathing and to make my body function, the jaw got locked to chew, drink, swallow; I had to start from zero (Emily).*

*Life teaching.* With respect to this subcategory, the participants reported being more empathetic with the types of diversities, being more aware of what you have and had at the time and of your own meaning in life: *I believe each day is a lesson and it is a staging, so to speak, to teach me about the situations; in this term, it is to demonstrate through your position and your actions everything that can be done (Martín); A lesson that you can never take anything for granted, there is always something more that can be done, any adversity helps you to realize that from each individual’s mistakes and strengths, for example in my case, I wasn't very attentive to my family, I always called them about everything, but I always had other priorities, on a Saturday party, Sunday with friends, during the week, so family was not a priority for me, rather I have priorities that just give me momentary happiness (Eric).*

### Category 5. Spirituality

With respect to spirituality, most of the participants considered that having some type of belief establishes a bond of hope; the following is a story that encompasses most of the feelings and emotions they experienced at the time of acquiring diversity: *You believe in the giving God, the loving one or many times the opposite, it happened to me that when the accident happened to me, I fought with God and I said to God, “why did you send me something this hard”?, if I am a good person, I don’t go out; my brother was the crazy one in the house, he would stay out all night and I was the good girl, I would say “God, but why did you send me this? Until one day I came back from an MRI, which they had done about a month later and I cried a lot. In the MRI, I mentally asked God to clarify the situation, right? It was very nice because in my room in the hospital room, people came from all the regions you wanted; it was strange, the Catholic, the Christian, the Evangelical went, they were people that I didn't even know, that is, I was amazed. I would say somehow God was talking to me through those people, so I said, no, I won’t fight with Him anymore, I will accept the situation, I will follow spirituality (Emily).*

### Category 6. Autonomy

Autonomy is, thus, intertwined with the freedom our participants have to decide on any aspect of their lives: *I decided to do absolutely nothing based on the others, I live my normal life. Obviously, it is much more complicated because I use a walker and some splints to move, I mean, I have to exert more force, everything is more complicated, but I prefer complexity than comfort (Eric).* Nevertheless, those interviewed report that often these decisions may be affected as they cannot develop independently and require support*: You are going to face processes where they are going to tell you to sit down, at the beginning of the process and you are not going to be able to sit down, or they are going to tell you, as in my case, that I was not going to move anymore, they had to do everything for me, grasping something; I have a problem with my hands, it's not very noticeable, but I couldn't hold anything, absolutely nothing, that is, my hands were shaking, the weight of anything was very difficult, but I always mentalized myself that I had to little by little get over it and not think about my diagnosis (Emily).*

### Category 7. Education

According to what was stated by the interviewees, it is a right that in the population with motor functional diversity is still violated by multiple barriers of access and permanence: *What happens is that for us there is more difficulty, starting with transportation, starting with job opportunities, with education, it's more difficult, it's a little more difficult, so things take a little bit longer. (Martha). The university’s facilities have many areas where, for example, ramps are needed; on a physical level, more part of inclusion and on an educational level it is very general and the capabilities of each person are not taken into account (Milagros).*

*Inclusive-education strategies.* During the entire interview, the participants described multiple strategies that could be implemented within the university campus, which would potentially help the adaptation and permanence of students with motor functional diversity: *All the strategies that can be used, whether they are sound, visual, expectation and motivation campaigns; I think they all work. What happens is that it is a process that does not happen overnight, it has to be accompanied by other processes, as well as family members, educational processes in institutions, social processes where one develops in other activities (Martín). I ask the university to have strategies; they have technology, internet, many devices, so promote virtual classes, when we cannot attend for something serious, give us the opportunity, because many teachers do not understand, it is a struggle, so they should take more into account that if we do not go it is not because we do not want to, but because of health, it is complicated for us (Martha).*

*Visibility.* The participants consider that when talking about this theme, the multiple problems faced by the population with functional diversity are being made visible, given that there is segregation and, to eliminate these injustices, it would be necessary to make visible, normalize and value the capacities and skills of those with diversity: *I believe that this, what you are doing is an issue that will leave the university with a process that they have to start implementing in some other way (Federico). I would think that it would be very good if inclusive education was promoted more, I would really like more people to study so that children who come with different conditions, have the dreams of looking at people, because they identify, achieve those dreams, yes? and to keep fighting, because that motivates, helps them grow and thank you for those spaces because how nice, how nice that you, as students, are empathetic, dream of a different, diverse, capable university; I like it, how good, how beautiful and I congratulate you for making this more enjoyable (Joaquín).*

*Advice.* Finally, during the interview, the participants were asked to provide some advice for future students to manage to adapt to the university campus in order to complete their academic process: *At the beginning, focus on what recovery is, focus a lot, and as one acquires skills and can develop academic life, then progress little by little and not get too saturated (Antonia). That they do not see their limitation as a barrier, as not an impediment, suddenly it is a barrier, in many things, in its literal form, but they do not see it as an impediment to developing their life project, because people with disabilities can do it, we can all finish the career, we can have an important life, we can have an important role in society, we can have a family, friends, children; it can be done, that they do not focus on the fact of having a limitation because that's it, you don't have to think about the hand but about what I can reach (Joaquín); and*, *To always look for a way to cope with everything, that there are always possibilities of absolutely nothing being done, one always has to do it, so it doesn't matter if you tell me it can't be done, why can't it be done? There will always be a way to achieve what you want as long as you want everything is achievable, there is no big or small obstacle, there are simply potholes that you always have to find a way to overcome and nothing else (Eric).*

## Discussion

This study, in the first place, observed that students with motor functional diversity found difficulties with the lack of inclusive architectural design in the university infrastructures because most of them are not fit for all, for example, restrooms, cafeterias, second- and third-floor classrooms, and some administrative areas; this result is similar to the study by Santos *et al.*^13^ As stated by Domínguez *et al*.,^14^ people with motor functional diversity can be independent, as long as they have the means of accessibility to achieve it. Secondly, Toutain^15^ indicates that the most-common barriers to adaptation are lack of knowledge by students about the resources of the campus and the inability to know the exact number of enrolled students who have a disability; similarly, Pérez *et al.,*^16^ mention in their systematic review that these were the difficulties most stated by the participants, which shows that this problem does not only occur in our country. 

This study also found non-compliance with legal norms to satisfy the minimum needs of people with disability, therefore, it must be emphasized that inclusive education is a project of political struggle requiring critical participation in the communities.^17^^,^^18^ Likewise Vargas^19^ highlights the need for research groups to address the issue of inclusion, creating possibilities to broaden the debate in universities and promoting an inclusive culture through seminars and continuous education courses, among others.

In third place, as indicated by Segers *et al.,*^20^ it was noted that the use of coping mechanisms fosters the capacity for resilience and are relevant to their personal development, allowing students to adapt to stressful situations and reducing the negative impact on their psychological wellbeing. Resilience is the principal characteristic developed by the participants in the present research and they state that it was the basis of their adaptation process, agreeing with the results obtained by Lozano.^21^

Fourth, as described by the participants, it is necessary for universities to implement inclusive-education strategies, such as recorded classes or live transmissions, highlighting the need to use CIT tools. As expressed by Fernández and Afirrano^22^ in their research “Technological Teacher Training and Disability”, these technologies are facilitating resources and transmitters of information, which can be adapted to the needs and characteristics of each student in personalized manner and represent an excellent alternative to achieve their inclusion in the society del knowledge, contributing to equity in access and to the quality of learning,^23^ with these authors highlighting that inclusion also means providing the necessary tools for success.^24^

Finally, nurses must be excellent caregivers, perceptive to states of wellbeing and health problems of individuals and must be able to offer care in different sociocultural contexts, using critical thinking and communication skills,^25^ given that they face different challenges daily, with each individual having diverse characteristics and needs, which merit personalized care; hence, knowing the coping strategies of students with motor functional diversity contributes to scientific knowledge, the discipline, and the professional practice, responding to future care needs and innovating within the educational environment. 

## Conclusion

The support and affection provided by the social setting plays a fundamental role in the adaptation of students with motor functional diversity, improving their mental health, generating resilience, and increasing their self-esteem. Noting that despite lifestyle changes after the acquisition of diversity, students set new goals and new skills that contribute to their life project; likewise, they can recognize their coping mechanisms, especially throughout their rehabilitation process, acquiring qualities, like autonomy.

The researchers, herein, recommend for universities to implement inclusive-education strategies, like recorded classes or live transmissions, in case students in general cannot attend due to health reasons and minimize physical barriers that prevent easy access to the campus, generating greater physical effort for them, thus, demonstrating that adjustments must be made to the infrastructure of the institutions to guarantee education for the entire population. In addition, as nurses, we entrust the construction of a nursing care model that contributes to the adaptation of students with motor functional diversity based on the information obtained in this research, thereby, contributing comprehensive care and promoting the discipline’s own knowledge.

## Annex. Interview guide instrument Adaptive Mode: Interdependence

1. How would you like for your loved ones to express feelings toward you?
2. What were the feelings you experienced when you acquired your functional diversity? How did you deal with them?
3. How do you express your feelings?
4. How do you realize that you are loved or liked by someone?
5. Do you believe that demonstrations of love are fundamental to adapt to change? Why?
6. What have been the demonstrations of love that have helped you to deal with change?
7. What situations provoke positive emotions in you?
8. What activities do you do to improve your mood?
9. How did you evidence support from your family?
10. What role does your family play at the moment of adapting? Why?
11. Did you get any economic aid from the state? What was it and how did you get it?
12. What strategies or activities did your social circle undertake as demonstration of support?
13. What significant teaching have you experienced that contributes to your adaptation process?
14. What goals have you set? How many of them have you achieved?
15. What changes have you made to reach your goals?
16. Do you think that you have grown as a person after acquiring the functional diversity? Why?

Adaptive Mode: Role Function

1. How has your life project changed? How did you deal with it?
2. What strategies did you use to rethink a new life project?
3. What coping strategies did you use to supply changes to your abilities?
4. Do you consider that acquiring a functional diversity altered your economic stability? How did you deal with it?
5. Have you found obstacles in your academic environment? Which, and how have you dealt with them?
6. What educational strategies do you think should be implemented to comply with an inclusive education?
7. Do you consider that the functional diversity affected the execution of the role in your family? How did you deal with such?
8. What role do you consider your family plays? Do you think it changed after acquiring the diversity? How did you deal with the change?
9. Do you consider that acquiring a functional diversity hinders being able to work? What coping strategies do you use?
10. Do you consider there was a change in your social circle after acquiring the functional diversity? What coping strategies did you use?
11. Do you think that acquiring diversity altered the development of your hobbies? How did you deal with that change?

Adaptive Mode: Self-concept

1. What were your emotions the moment of perceiving the change in your bodily sensation? What coping strategies did you use?
2. Have you had access to physical therapy? (Yes) Which? (No) Why?
3. Do you consider that physical therapy contributes to your adaptation? Why?
4. Has the acquisition of the functional diversity affected your self-esteem? Why?
5. How have you managed to increase self-esteem?
6. What activities have you carried out to increase your self-esteem?
7. What decisions do you consider were fundamental for the adequate process of your adaptation?
8. What strategies did you use to carry out an adequate decision-making process?
9. Do you consider that your future expectations changed after acquiring the diversity? How? What strategies did you use to deal with it?
10. Which of the values inculcated do you consider to have been fundamental in adapting to the situation? Why?
11. Do you consider that religion or spirituality played an important role in your adaptation process? Why?
